# MAP-box: a novel, low-cost and easy-to-fabricate 3D-printed box for the storage and transportation of dissolving microneedle array patches

**DOI:** 10.1007/s13346-023-01393-w

**Published:** 2023-07-21

**Authors:** Qonita Kurnia Anjani, Álvaro Cárcamo-Martínez, Luki Ahmadi Hari Wardoyo, Natalia Moreno-Castellanos, Akmal Hidayat Bin Sabri, Eneko Larrañeta, Ryan F. Donnelly

**Affiliations:** 1 School of Pharmacy, Queen’s University Belfast, 97 Lisburn Road, Belfast, BT9 7BL Northern Ireland UK; 2 Boehringer Ingelheim Pharma GmbH & Co. KG, Biberach an Der Riss, Germany; 3 Fakultas Seni Rupa Dan Desain, Institut Teknologi Bandung, Jl. Ganesa No.10, Bandung, 40132 Indonesia; 4 Basic Science Department, Faculty of Health, Universidad Industrial de Santander, Bucaramanga, 680001 Colombia

**Keywords:** MAP-box, Microarray patches, 3D-printing, Packaging, Donepezil, Stability

## Abstract

Research on the use of microarray patches (MAPs) has progressed at an unprecedented rate over the years, leading to the development of many novel drug delivery systems. As the technology approaches patients, there are several key aspects that ought to be addressed in order to facilitate the smooth translation of MAPs from bench to bedside. One integral factor includes the choice of devices and packaging for the storage of MAPs. In the current work, a slide-and-seal box, MAP-box, was developed for the storage of dissolving MAPs, using fused-deposition modelling. The device has been designed to act as a pill-box for MAPs not only to provide protection for MAPs from the environment, but also to improve patient’s adherence to treatment. The overall design of the MAP-box was simple, yet offers the capability of sealing and protecting dissolving MAPs up to 30 days. Donepezil HCl was formulated into a dissolvable MAP, which was used to treat dementia related to Alzheimer’s disease. This compound was used as a model formulation to evaluate the utility of the 3D printed MAP-box when placed under three storage conditions: 5 °C and ambient humidity, 25 °C and 65% relative humidity and 40 °C and 75% relative humidity. It was shown that the slide-and-seal box was able to confer protection to MAPs for up to 30 days under accelerated stability study conditions as the drug loading, mechanical properties and insertion properties of MAPs remained unaffected when compared to the unpackaged MAPs stored under these same parameters. These preliminary data provide evidence that the MAP-box prototype may be of great utility for the storage of single or multiple MAPs. Nevertheless, future work will be needed to evaluate their patient usability and its application to different types of MAP systems to fully validate the overall robustness of the prototype.

**Graphical Abstract:**

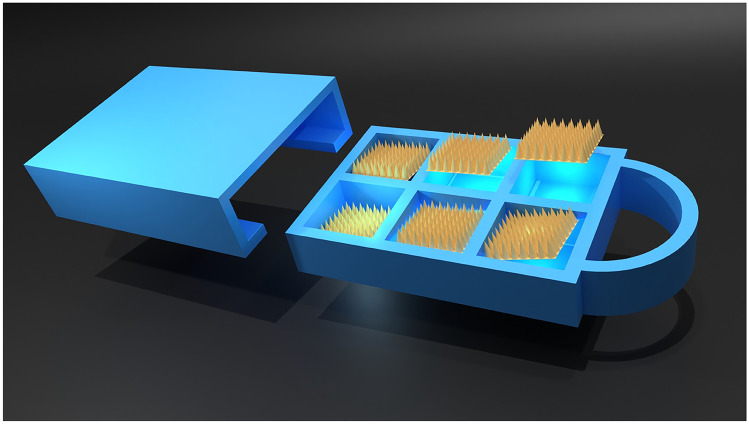

## Introduction

Transdermal drug delivery has attracted much-increased attention over recent years due to the invention of microneedle array patches (MAPs), which are very small needles able to pierce the *stratum corneum,* the outermost layer of the skin. Since the active substance is often incorporated in/on the needles, this system can effectively deliver payloads into the skin and/or, later on, the systemic circulation. By doing so, MAPs can overcome the limitations of conventional transdermal patches and the physicochemical requirements of the compounds included, such as log *P* value between 1 and 3, high potency and lipophilicity, low molecular weight (< 600 Dalton) and low melting point (< 250 °C) [1].

What started as an academic endeavour has now attracted the attention of multiple pharmaceutical companies globally, looking into a variety of applications for such drug delivery devices. For instance, Sanofi Pasteur registered an intradermal influenza vaccine under the name of Intanza^®^ in 2009 in Europe, which involved a 1.5-mm length hollow metallic microneedle (previously developed and registered by Becton–Dickinson) [2]. Similarly, Zosano Pharma developed a titanium-made microneedle system coated with zolmitriptan for the treatment of migraine [3]. Although the FDA denied the marketing authorization of this new drug application, leading to the bankruptcy of the company in 2022, their extensive clinical work suggested that the first drug product approved, as a whole MAP device, for drug delivery is likely to be far away. Additional examples of industrial interest in this technology are those of LTS Lohmann Therapie-Systeme AG and Corium Inc, who already hold manufacturing licenses for the fabrication of microneedle array patches under standards of Good Manufacturing Practice (GMP) [4].

To date, however, there is no clear guideline on the critical quality aspects to be evaluated, nor how this should be done, to prepare a common technical document for the submission of MAPs as drug products to regulatory agencies. This is further complicated by the fact that there are different types of MAPs with different drug delivery modes. Briefly, MAPs can be classified as either solid, dissolving, hollow, hydrogel-forming and coated [5]. As MAPs represent a painless, self-administered alternative for the administration of drugs, they can be a valuable option for the treatment of diseases in developing-world countries [6]. Hence, their transportation and storage will be critical steps, even more considering the diverse temperature/humidity conditions in those countries. On that note and due to their simplicity, dissolving MAPs are one of the most reported in literature for potential applications in vaccination, HIV/AIDS prevention/treatment and for the delivery of a wide range of drugs [7–12]. However, as this class of microneedles are typically fabricated from either polymers [9] and/or sugars [10] that are hygroscopic, the exposure of these MAPs to the atmosphere may increase the propensity of the patches to entrap moisture into the needles [13, 14]. The presence of excessive moisture in these needles would result in plasticisation along the needle length, which would weaken the overall mechanical properties of MAPs, resulting in poor insertion into the skin [15]. In addition, the exposure of MAPs to high temperature and sunlight may also increase the likelihood of payload degradation, as well as affecting the mechanical properties of the needles during storage that could result in sub-therapeutic drug doses being delivered upon administration [16]. Since MAPs should be inserted into the skin successfully and consistently for optimal and reproducible drug or vaccine delivery, their insertion ability over time would indeed be a critical quality aspect to be tested. Similarly, the resistance of needles to compression would be an indicative of whether they will be able to withstand the compressive force needed for their insertion after storage [17]. Therefore, the design of appropriate storage solutions for dissolving MAPs is without a doubt a critical parameter that ought to be considered and investigated as the technology progresses from the lab to the clinic.

Pill-boxes have been used regularly to store and ease pill administration in different types of patients [18]. Their use has been successfully implemented for elderly patients that require regular administration of multiple types of tablets [19]. These boxes contain several compartments to place daily oral medication so patients can organise their pill burden and track their uptake. Therefore, we believe that it can be applicable for MAP technology. However, its application to MAP technology requires the evaluation of alternative parameters such as the mechanical integrity of the MAP arrays during storage and their protection from environmental conditions. The latter is especially important as MAPs will be taken out from their original packaging to be placed into these boxes. These boxes can be used by patients in different parts of the world under different conditions and therefore the device should provide protection to the MAP arrays.

In this study, we developed and evaluated a 3D-printed MAP-box similar to a conventional pill-box using fused-deposition modelling for storing dissolving MAPs. The box is designed as a slide-and-seal, providing user-friendly functionality. We selected Alzheimer’s disease as a good model as patients suffering from this condition have medication adherence problems. A “MAP-box” could help these patients with adherence to treatment. Therefore, we prepared MAP loaded with donepezil hydrochloride (HCl), which is commonly used to treat Alzheimer’s disease-related confusion (dementia) [20]. While donepezil HCl does not cure or alter the course of the disease, it has been shown to improve memory, awareness and functional abilities [21]. Oral administration is the typical route for delivering Donepezil HCl. However, this method often leads to gastrointestinal side effects such as nausea, vomiting, anorexia and diarrhea, potentially hindering patients from receiving effective doses [22]. Dysphagia, which becomes more prevalent as the disease progresses, further necessitates exploring alternative administration routes [23]. Dissolving microneedles offer a potential solution for delivering antidementia drugs like donepezil HCl, aiming to improve treatment and care for patients with Alzheimer’s disease. Our 3D-printed MAP-box facilitates storage and assists elderly patients and caregivers in tracking the number of applied patches, reducing the risk of missed doses. Additionally, the low cost and ease of manufacturing make this box suitable for mass vaccination and treatment in developing countries. These factors are important considerations as we advance towards the translation and commercialisation of MAPs.

## Materials and methods

### Materials

Donepezil HCl was obtained from Tokyo Chemical Industry UK Ltd. (Zwijndrecht, Belgium). Polymers poly(vinyl alcohol) (PVA, MW 9,000–1,0000 Da) and Plasdone^®^ poly(vinylpyrrolidone) (PVP, MW 58,000 Da) were purchased from Sigma-Aldrich (Dorset, UK) and Ashland Industries Europe GmbH, Switzerland, respectively. Parafilm^®^ M used for simulating skin for insertion studies was purchased from Bemis. Other chemicals, salts, buffer tablets and analytical and HPLC-grade solvents were purchased from Sigma-Aldrich (Dorset, UK). Ultrapure water was obtained from a water purification system Elga PURELAB DV 25, Veolia Water Systems (Dublin, Ireland). Full-thickness neonatal porcine skin was obtained from stillborn piglets < 24 h after birth, rinsed in PBS and kept frozen at − 20 °C until use.

### Fabrication of MAPs

Dissolving MAPs were fabricated using a PDMS mould with the following design: 600 pyramidal needles on a 0.76 cm^2^ area with 750 µm needle height, which were fabricated as previously described [12, 14, 24]. Polymeric MAPs were fabricated by using a two-step micromoulding procedure. First, 150 mg of donepezil hydrochloride was mixed with 150 mg of aqueous polymeric solution (containing 20% w/w PVA and 20% w/w PVP), and 300 mg of deionised water was mixed before the blend was cast into the moulds, which were then subjected to a positive pressure chamber with a pressure of 5 bar for 5 min. Excess blend on the moulds’ surface was meticulously removed before the moulds were centrifuged at 5000 rpm for 10 min. Then, moulds were dried under ambient conditions overnight before a baseplate containing 30% w/w PVP (MW 90 kDa) and 1.5% w/w glycerol was cast on top of the needle layer and centrifuged at 3500 rpm for 15 min. The system was left to dry overnight and demoulded the next day with excess baseplate being meticulously trimmed off. Prior to any further characterisation, MAPs were dried again at 37 °C for 24 h.

### Height reduction

A Texture Analyzer TA.XT-Plus (Stable Microsystems, Surrey, UK) was used to evaluate the resistance to compression of dissolving MAPs, as previously described [14, 25]. The instrument was set in compression mode (pre-test speed: 1 mm/s, test speed: 1 mm/s and post-test speed: 1 mm/s) and using a force of 32 N per 30 s. Needles’ height was recorded prior to and immediately after the test using a light microscope (Leica EZ4 D, Leica Microsystems, Milton Keynes, UK).

### MAPs insertion in skin models

Insertion of MAPs was tested using both Parafilm^®^ and full-thickness neonatal porcine skin as models [12, 26]. In brief, Parafilm^®^ stacks (8 layers in total) were positioned on the metallic base of the Texture Analyser while MAPs were attached to the upper mobile probe. The equipment was set in compression mode (pre-test speed: 1 mm/s, test speed: 1 mm/s and post-test speed: 1 mm/s), the force was 32 N and the test was run for 30 s. After the experiment, Parafilm^®^ layers were collected and evaluated with a light microscope, and the number of pores generated was evaluated and calculated using Eq. 1:

1 $$\%\; \mathrm{holes}\; \mathrm{generated}=\frac{Number\; of\; pores\; visualised}{Number\; of\; needles\; per\; array^*}\times 100$$

*the number of needles per array was 600

To complement the insertion study into Parafilm^®^M, an additional penetration test using full-thickness ex vivo neonatal porcine skin was conducted using the same setup. Then, the penetration depth of needles was measured by observing the skin with an optical coherence tomography (OCT) microscope (EX1201, Michelson Diagnostics Ltd., Kent, UK), as previously reported [27].

### Calculation of drug content

Donepezil-loaded dissolving MAPs were dissolved in separate scintillation vials containing 5 mL of deionised water and using an ultrasonication bath (The Ultrawave U500H, West Bromwich, UK) for 30 min. The mixture was diluted by the addition of 5 mL of methanol, followed by another cycle of sonication for the same period. The samples were then centrifuged at 14,500 rpm for 15 min before being taken for HPLC analysis.

### Ex vivo skin dissolution studies

Similar to the insertion protocol in “MAPs insertion in skin models”, the dissolving MAPs were manually inserted in full-thickness neonatal porcine skin using thumb pressure for 30 s. Next, a cylindrical weight (circa 15.0 g in weight) was placed on top of the patch to prevent expulsion of the patch following insertion. Dissolving MAPs were then stored at 37 °C. At defined time points, MAPs were carefully removed, and the remaining needles were visualised under a light microscope.

### Ex vivo skin deposition studies

Skin deposition studies were conducted in a similar fashion as previously reported [14, 28], using 12 mL vertical Franz diffusion cells (PermeGear, Inc., Hellertown PA, USA). Briefly, MAPs were applied to full-thickness porcine skin which had been secured to the donor compartment using cyanoacrylate glue. The insertion was performed by pressing MAPs using thumb pressure for 30 s. Upon inserting the MAP into the skin, the Franz cell was assembled by attaching the receiver compartment to the donor compartment filled with degassed phosphate buffer saline pH 7.4. In addition, a cylindrical metal weight was placed above each MAP to prevent patches from being dislodged from the skin during the permeation study. At predetermined time points, the cells were disassembled, and the receiver compartment and skin samples were collected for processing and analysis. The collected skin samples were placed on a heating mantel set to 60 °C to promote the separation of the epidermis from the dermis. Upon separation, the epidermis was placed in an Eppendorf tube containing 2 mL of methanol, as an organic solvent to extract donepezil, with two stainless steel beads (5 mm diameter). The samples were homogenised for 30 min (ThermoMixer™F2.0, Eppendorf, Hamburg, Germany). In addition, the dermis was homogenised using 500 µL of deionised water for 15 min before being subjected to further homogenisation for 15 min following the addition of 1 mL of methanol. All homogenisation steps were performed at 50 Hz. All samples, including the receiver compartment contents, were centrifuged at 15,300 rpm for 15 min, and the supernatants were collected for HPLC analysis.

### MAP-box design

**Fig. 1:**
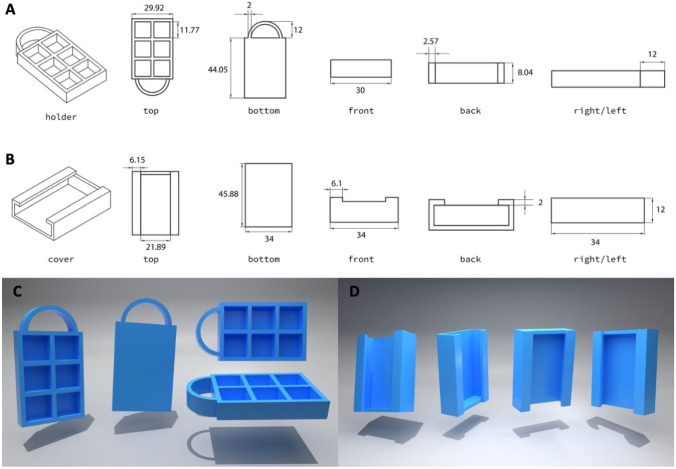
Two-dimensional drawing of designs at different perspectives. **A** MAP-box and **B** the cover. The dimensions are shown in mm unit. CAD designs of MAP-box at different perspectives. **C** MAP-box and **D** the cover

**Fig. 2:**
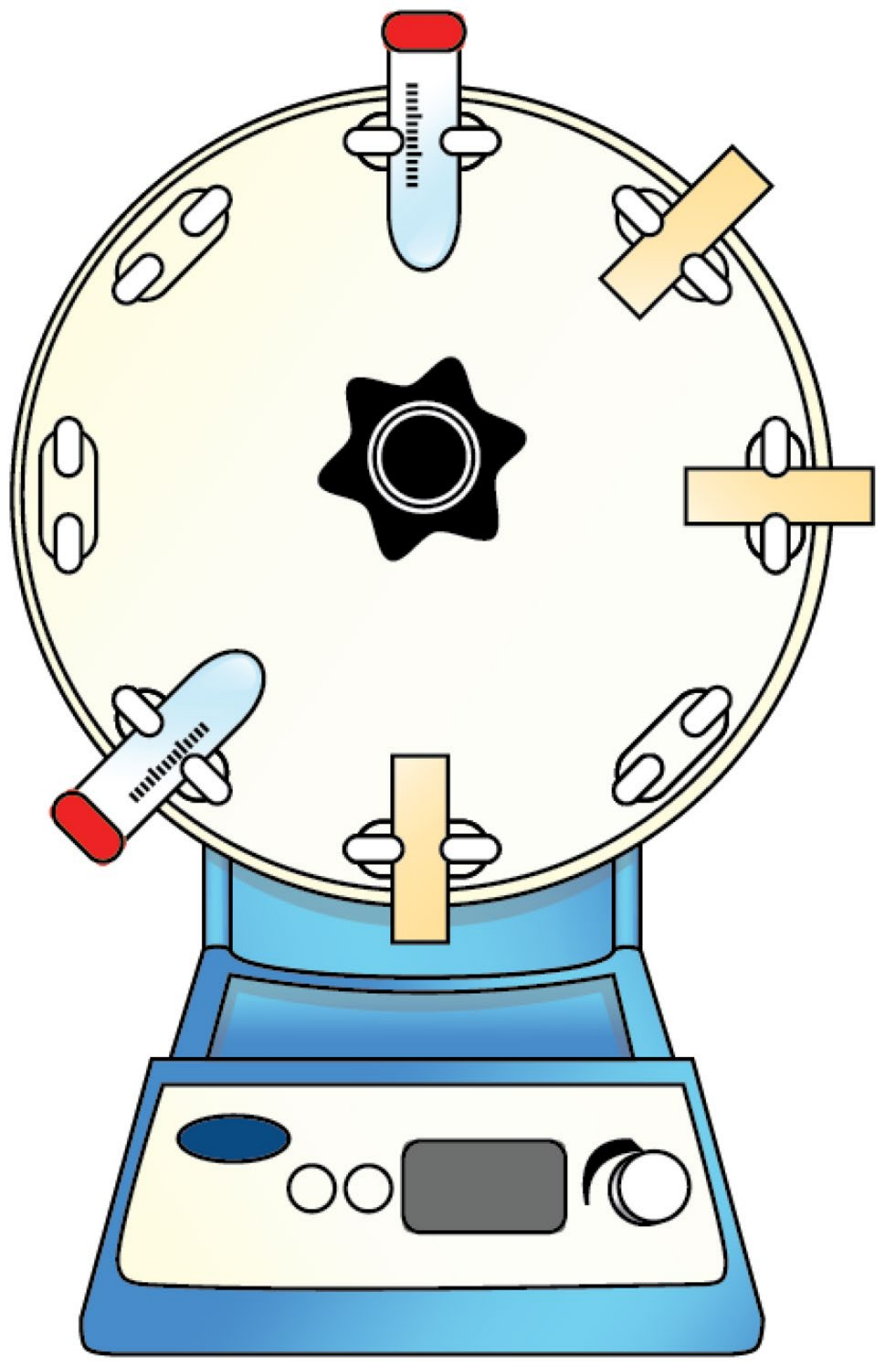
Illustration of the setup for the friability test

The MAP-box was designed using Tinkercad^®^ software and printed using a fused deposition modelling (FDM) 3D printer (Ultimaker 3, Ultimaker, Zaltbommel, Netherlands) with a 0.4-mm print core. The material and key printing parameters for 3DP are as follows: poly(lactic acid) filament (PLA, 2.85 mm diameter, pearl white, Ultimaker), 100% infill, 0.1 mm layer height, 0.38 mm line width, 205 °C printing temperature, 85 °C build plate temperature and 25 mm/s print speed. As shown in Fig. 1, the dimensions of the MAP-box were 5 cm in height, 3.5 cm in width and 1 cm in depth. Within the MAP-box, there were 6 individual boxes with an area of 1 cm^2^ for the storing of MAPs. In addition, the computer-aided design (CAD) images of the MAP-box are shown in Fig. 2.

### Morphology and structure of the MAP-box

Both surface and morphology of the MAP-box were examined using a TM3030 scanning electron microscope (SEM) (Hitachi, Krefeld, Germany). To analyse the samples, a 1 cm × 1 cm flat square was printed using the same method as the MAP-box and placed on adhesive carbon tape. Additionally, the MAP-box structure’s cross-section was visualised using an X-101 optical coherence tomography (OCT) microscope (Michelson Diagnostics Ltd., Kent, UK).

### Moisture permeability of the MAP-box

To investigate the protective effect of the designed MAP-box against moisture, silica gel was weighed and placed into both open and closed MAP-boxes. The MAP-boxes were then exposed to an accelerated condition of 40 °C and 75% relative humidity for 1, 2 and 7 days. The weight of the MAP-boxes containing silica was recorded at predetermined time points to evaluate the level of moisture absorption.

### Water contact angle of the MAP-box

The contact angle between the surface of the MAP-box and deionised water was measured using an Attension Theta optical tensiometer (Biolin Scientific, Gothenburg, Sweden). The sessile drop method was used to determine the contact angle, both before and after storing the MAP-box in a stability cabinet at 40 °C and 75% relative humidity. A 4 µL droplet of water was placed on the MAP-box’s surface, and the contact angle was measured 30 s after releasing the droplet. OneAttension software was used to analyse the results.

### Friability test of MAP inside the MAP-box

To assess the mechanical resistance of needles inside the MAP-box during transportation, four MAPs were subjected to three different conditions. The first condition involved placing the MAPs inside a 50-mL falcon tube without a MAP-box. In the second condition, the MAPs were placed inside the MAP-box, while in the third condition, the MAPs were placed inside the MAP-box and secured with double-sided tape. The setup is illustrated in Fig. 2. The containers were then attached to a rotator (Stuart^®^ SB3 Rotator, Davidson & Hardy Ltd., Dublin, Ireland) and rotated at 40 rpm for 1 h. The needle height of each MAP was measured before and after rotation, and the results were compared.

### Stability studies

To evaluate the protective effect of the designed MAP-boxes, donepezil-loaded MAPs were exposed to the following three conditions: 5 °C and ambient humidity, 25 °C and 65% relative humidity and 40 °C and 75% relative humidity for 14 and 30 days. In all cases, MAPs were placed inside the containers before exposure to such conditions, using unpacked MAPs as controls. At the defined time points, drug content, reduction in height of needles and insertion depth (using full-thickness porcine skin) were evaluated, similarly as described in the previous sections.

### High-performance liquid chromatography analysis

The amount of donepezil HCl was measured using HPLC (Agilent Technologies 1220 Infinity UK Ltd., Stockport, UK). A XSelect CSH C18 column was used to separate the analytes, with a 3.0 mm internal diameter, 150 mm length, 3.5 µm particle size and a pore size of 130 Å (Waters, Dublin, Ireland). Prior to the column, a VanGuard^®^ cartridge (3.9 mm internal diameter, 5 mm length) was used (Waters, Dublin, Ireland). Sample analysis was conducted at a flow rate of 0.6 mL/min and 30 °C. The mobile phase was a mixture of 0.1% v/v trifluoroacetic acid in water and acetonitrile in a ratio of 60:40 v/v. Detection was done at 235 nm, and 20 µL was used for sample injection. The HPLC method used in the study was validated in accordance with the International Council on Harmonisation (ICH) 2005 guideline.

### Statistical analysis

GraphPad Prism^®^ version 8.0 (GraphPad Software, San Diego, California, USA) was used to analyse all collected data. The results are presented as means ± standard deviation (SD), unless otherwise specified. Student’s *t*-test was used to determine significant differences between two groups, while one-way analysis of variance (ANOVA) was used to compare differences between multiple groups of data. Statistical significance was considered at *p* < 0.05.

## Results and discussion

### Fabrication and characterisation of MAPs

**Fig. 3:**
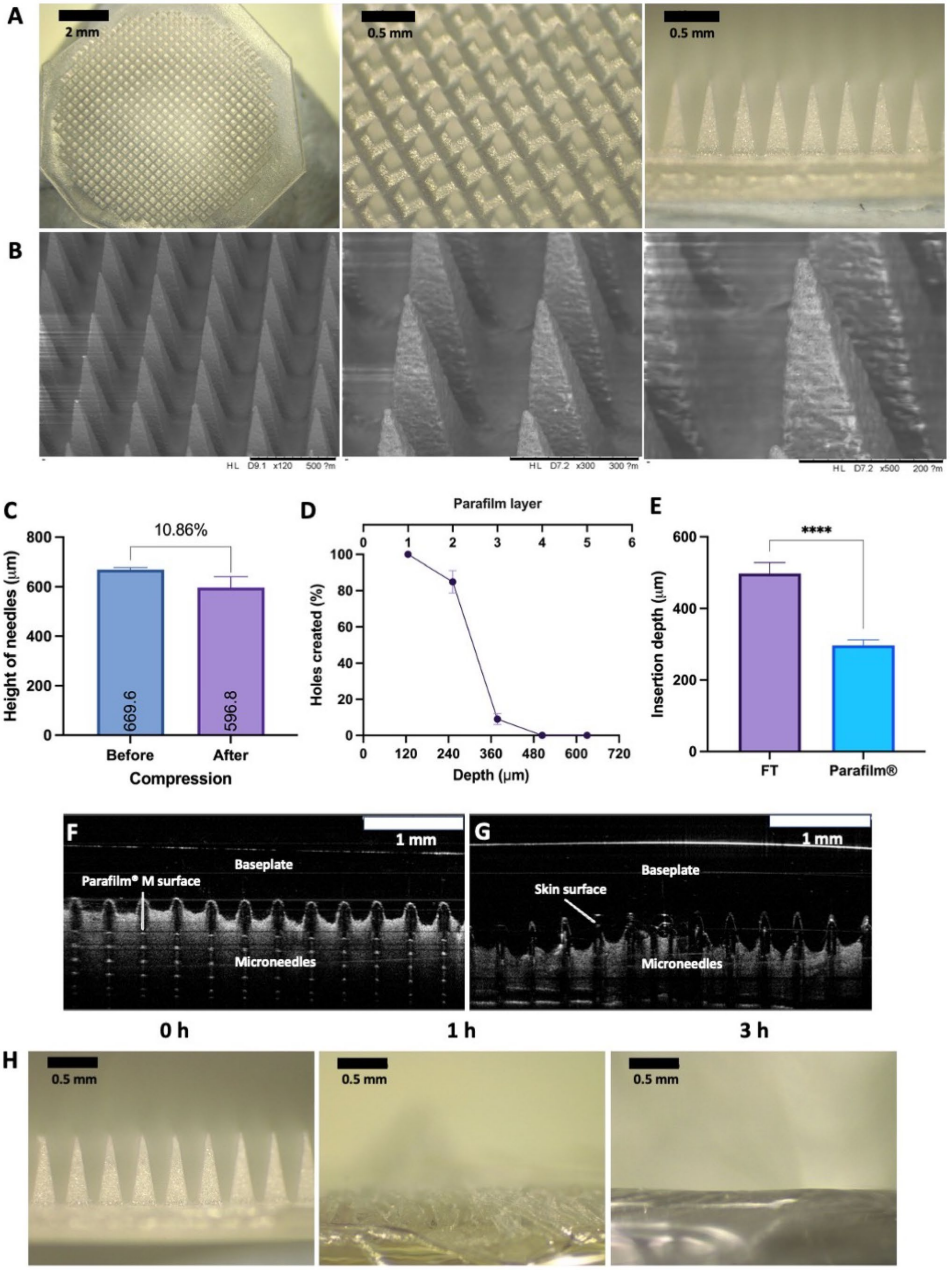
Optical characterisation of donepezil HCl-loaded MAPs. **A** Light microscopy images of MAPs. **B** SEM images of MAPs. **C** Reduction in needles height after being compressed for 30 s with a 32 N force (means + SD, *n* = 20). Insertion capabilities of donepezil-loaded MAPs into **D** Parafilm^®^M (measured by holes created in each layer) (means ± SD, *n* = 3), Parafilm^®^M and full thickness (FT) porcine skin (measured by OCT) (means + SD, *n* = 20). Representative OCT images of MAPs insertion into **F** Parafilm^®^M and **G** full-thickness porcine skin. **H** In situ skin dissolution of donepezil-loaded MAPs at defined time periods

A two-step casting procedure was adopted in the manufacture of donepezil-loaded dissolving MAPs. It can be seen that, post-fabrication, the patches displayed uniform arrays of microprojections on a smooth and flat baseplate. In addition, it is apparent that there were no observable signs of air bubbles or unevenly formed needles, as shown in Fig. 3A and B. The ability to manufacture dissolving MAPs in a reproducible way confirms that the use of a pressure chamber in tandem with centrifugation offers a viable approach to produce the dissolving MAPs.

Following the fabrication of donepezil-loaded dissolving MAPs, the patches were then subjected to a series of characterisation experiments in order to evaluate the properties of the patch. As it can be seen in Fig. 3C, the initial needle height was 669.6 µm. After being compressed with an axial force of 32 N, it can be seen that the needle height was reduced by approximately 10%. This reduction percentage in needle height following compression is similar to that previously reported for dissolving MAPs [12, 14]. This would indicate that MAPs would possess sufficient mechanical strength needed to withstand compression during skin insertion. To further evaluate this hypothesis, a set of insertion studies using a skin simulant, consisting of stack of Parafilm^®^M, and full-thickness ex vivo neonatal porcine skin were conducted.

As shown in Fig. 3D, the % holes generated across the stack of Parafilm^®^M decreases within increasing Parafilm^®^M layers. It was apparent that all the microprojections were able to pierce the first layer of Parafilm^®^ with no major variability between the replicates. Subsequently, approximately 80% of the needles on the array were able to pierce the second layer while less than 20% of the needles were able to pierce the third layer. There was no observable insertion generated within the fourth and fifth layers. When the insertion of MAPs in Parafilm^®^M was measured with the OCT, as shown in Fig. 3F and G, an insertion of ≈300 µm was seen, which is in agreement with the data shown in Fig. 3E. In contrast, when the study was performed on full-thickness porcine skin as shown in Fig. 3G, a significant higher insertion depth (*p* < 0.05) of 500 µm was achieved as shown in Fig. 3E. This could be a consequence of the nature of the skin models used, as Parafilm^®^ stacks are rigid and do not represent the elastic nature of the skin [17]. In addition, as the needle shafts of the MAPs are fabricated from water-soluble polymers, PVA and PVP along with the BCS class I drug, donepezil hydrocholoride, this translates into a very hydrophilic system. The insertion of the hydrophilic array into the skin, which is intrinsically rich in moisture due to the presence of interstitial fluid, could provide some level of lubrication to promote the insertion of the needles to a deeper extent into the skin relative to Parafilm^®^M, which is void of any moisture [12]. This discrepancy in insertion profile between Parafilm^®^M and ex vivo skin has been previously reported when evaluating the insertion profile of dissolving and hydrogel-forming MAPs [14, 25, 28].

The skin insertion studies were also complemented with an ex vivo skin dissolution study which sought to evaluate the duration and time taken for MAPs to fully dissolve upon administration into the skin. As shown in Fig. 3H, following 1 h of skin application complete dissolution of the needle layer of the MAP was seen, which indicated that the drug-loaded layer of the patch has been fully delivered into the skin forming an intradermal drug-polymeric depot. However, should the patch be left on the skin for up to 3 h, we observed complete dissolution of the entire patch, including the polymeric baseplate, which forms an occlusive and sticky polymeric gel on top of the skin surface. With regard to skin application, it can be concluded that the developed donepezil HCl-loaded dissolving MAP will only require an application time of approximately 1 h to enable complete dose delivery into the skin.

### Ex vivo skin deposition studies

Following the physical and mechanical characterisation of dissolving MAPs, the ability of the system to dissolve in the skin and deliver the drug across it was evaluated using a Franz cell diffusion setup. Prior to the permeation study, the drug loading of donepezil within MAPs was quantified by dissolving the MAP and quantifying it via HPLC. It was found that a MAP patch with an area of 0.76 cm^2^ containing 600 needles has a drug loading of approximately 3.31 ± 0.62 mg.

**Fig. 4:**
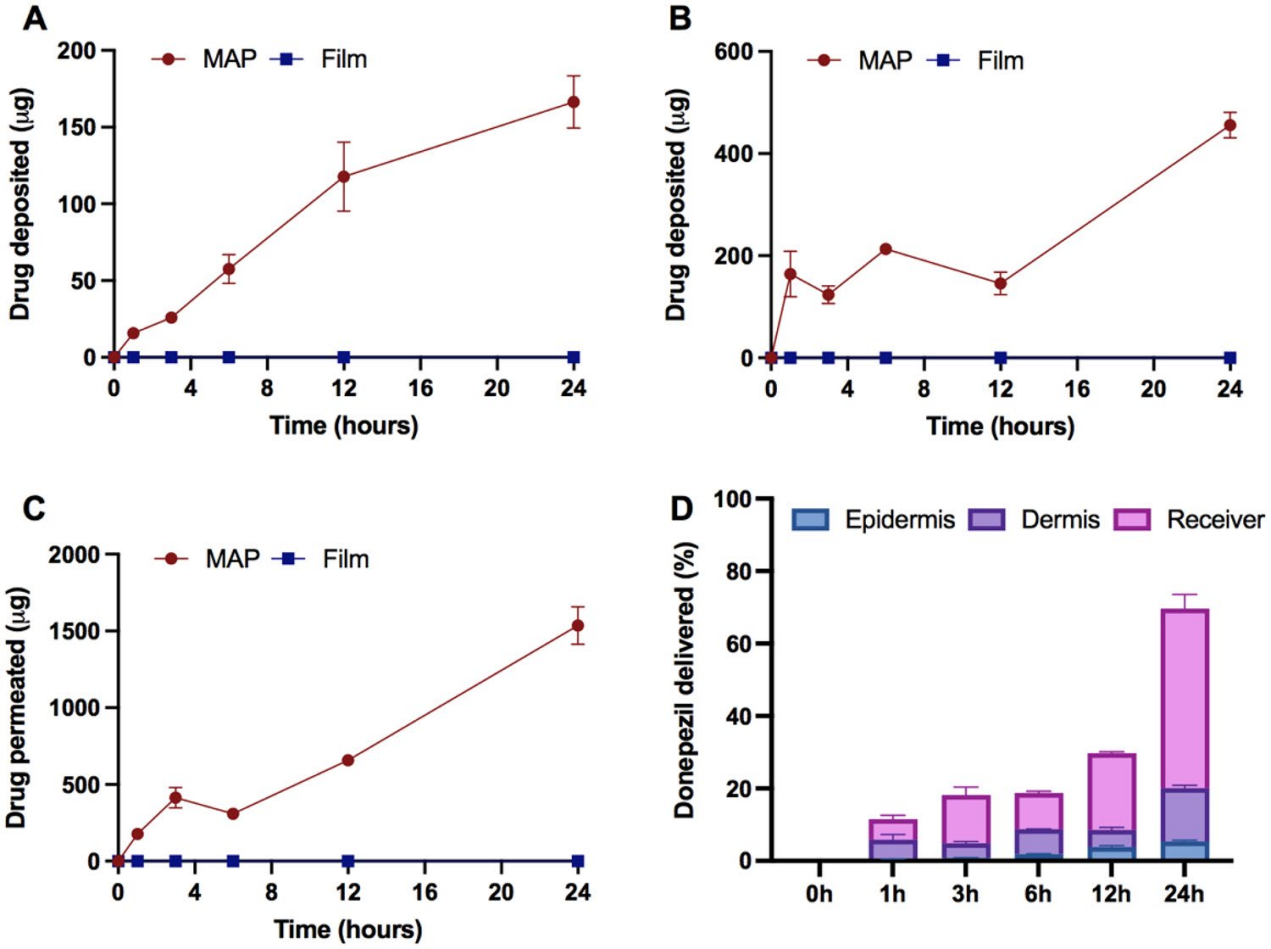
Skin deposition study of donepezil-loaded MAPs and film (as control) using full-thickness porcine skin. **A** Drug deposited in the epidermis (means ± SD, *n* = 6), **B** drug deposited in the dermis (means ± SD, *n* = 6) and **C** drug permeated to the receiver compartment (means ± SD, *n* = 6). **D** Overall donepezil released from MAPs over a course of 24 h (means + SD, *n* = 6)

Upon application to the skin, the permeation and deposition of donepezil across the different skin strata were assessed and quantified at defined time points. In addition, a thin film of donepezil hydrochloride that is void of any microneedles was used as a control. As it can be seen in Fig. 4A–C, no deposition of donepezil in the epidermis was seen when the film was used, while MAPs led to a sustained increase of drug deposition into the epidermis, dermis as well as the receiver compartment over the course of 24 h. This clearly indicates that formulating the active pharmaceutical ingredient (API) into a dissolvable MAP-based system provided the physical permeation enhancement needed to deliver the drug substance across the skin. It can be seen that almost within 1 h of MAP application, we observed levels of donepezil being delivered across the and continuously increasing with time. With regard to the level of donepezil being delivered across the different strata of the skin, we observed significantly higher drug being deposited into the dermis relative to the epidermis. This observation may be attributed to a combination of factors. First, the dermis is physiologically thicker relative to the epidermis, and therefore, it could enable more space for drug deposition [29]. Second, the intrinsically higher water content of the dermis relative to epidermis could favour the diffusion of the hydrophilic drug donepezil into the moisture-rich layers, as this would enable more of the drug to be dissolved and deposited [30]. It should also be noted that, due to the hydrophilicity of donepezil hydrochloride [31], it was observed that the majority of the drug diffused across the skin upon MAPs application and into the receiver compartment, culminating in an overall delivery of approximately 1500 µg after 24 h. Besides that, when the overall drug deposited is view as percentage delivered, it can be seen that the delivery efficiency of the patch increases over time as shown in Fig. 4D reaching an overall delivery efficiency of around ≈75% at 24 h.

It should be noted that several other researchers have also investigated the utility of MAPs as a potential drug delivery platform for the administration of donepezil. For instance, Kearney et al. [32] utilised hydrogel-forming MAPs prepared from Gantrez S-97, PEG, 10,000 and Na_2_CO_3_ in tandem with donepezil HCl-loaded films [32]. In contrast to the dissolvable MAPs developed in the current work, the delivery of donepezil using hydrogel-forming MAPs was achieved using a different mechanism of delivery. Upon application to the skin, the MAP could imbibe interstitial fluid which would then generate a continuous hydrogel network. By placing the drug-loaded film on the backing of the hydrogel-forming MAP, donepezil was able to diffuse from the drug-loaded reservoir across the swollen hydrogel network and into the skin, being then transdermally delivered. When evaluated in vitro using a Franz cell setup, researchers discovered that the system was able to achieve up to 0.85 mg of donepezil HCl delivered across the skin from the drug-loaded reservoir. In comparison, the dissolvable MAP developed in the current work was able to achieve almost twice the amount of donepezil delivered transdermally, 1.50 mg. Such lower amount of donepezil delivered using the hydrogel-based MAP may be attributed to the incomplete dissolution of the drug-containing reservoir, as well as a result of some of the drug being entrapped and deposited in the MAP itself [25]. It should be acknowledged that according to the British National Formulary (BNF), donepezil hydrochloride is given at a starting dose of 5 mg daily for the management of mild to moderate dementia in Alzheimer’s disease with an increase in dose of up to 10 mg daily, if necessary [33]. Therefore, based on the drug loading achieved and the established clinical-dose regime, it could be postulated that the administration of only two MAP patches developed in the current work would provide an equivalent starting daily dose for donepezil treatment. For the subsequent dose, the number of patches that needs to be applied could be increased for up to three (per administration). Nevertheless, should three patches be deemed too much, increasing the MAP size to enable large dose loading may serve as an alternative solution to limit the number of patches that needs to be applied. In this instance, the MAP area would need to be increased from 0.76 to 2.30 cm^2^ in order to achieve a drug loading of 10 mg per patch. A MAP with an area of 2.30 cm^2^ is approximately the size of a postage stamp and significantly smaller when compared to transdermal patches, which would enable the patient or career to administer the system under thumb pressure with ease.

In addition to the above, Kim et al. [34] developed a similar system to the current work, a dissolving MAP-based delivery system for the administration of donepezil. Unlike the current work, the drug loading for the system was much lower, as approximately only 180 µg of donepezil was found per patch. The MAP had an area of 0.56 cm^2^ and contained 100 microneedles per patch [34]. The lower drug loading used in the system developed by Kim and co-workers enabled most of the drug to be deposited within the tips of needles, which were fabricated from hydroxy-propyl-methylcellulose. When evaluated in ex vivo studies using porcine skin, the researchers were able to achieve a delivery efficiency of nearly 100% when applied for just a period of 5 min. Indeed, the duration of application needed to achieve complete dose delivery was far quicker than the one achieved in the present work. However, this short duration of application achieved is at the expense of the considerably lower drug loading, which was circa 18 times lower.

### MAP-box design

Following the extensive characterisation of the donepezil-loaded dissolving MAPs, the stability of the pharmaceutical system upon storage was evaluated in tandem with the use of a MAP-box prototype. In the current work, one of the main aims of the study was to develop a device similar to a pill-box which would enable a simple way to store MAPs while ensuring the stability of the formulation over defined time periods. The use of pill-boxes has been successfully applied to improve adherence to treatment in Alzheimer’s patients [35]. Therefore, this approach can be potentially used not only to protect MAP from physical stress and environmental conditions but to improve patient’s adherence to treatment.

**Fig. 5:**
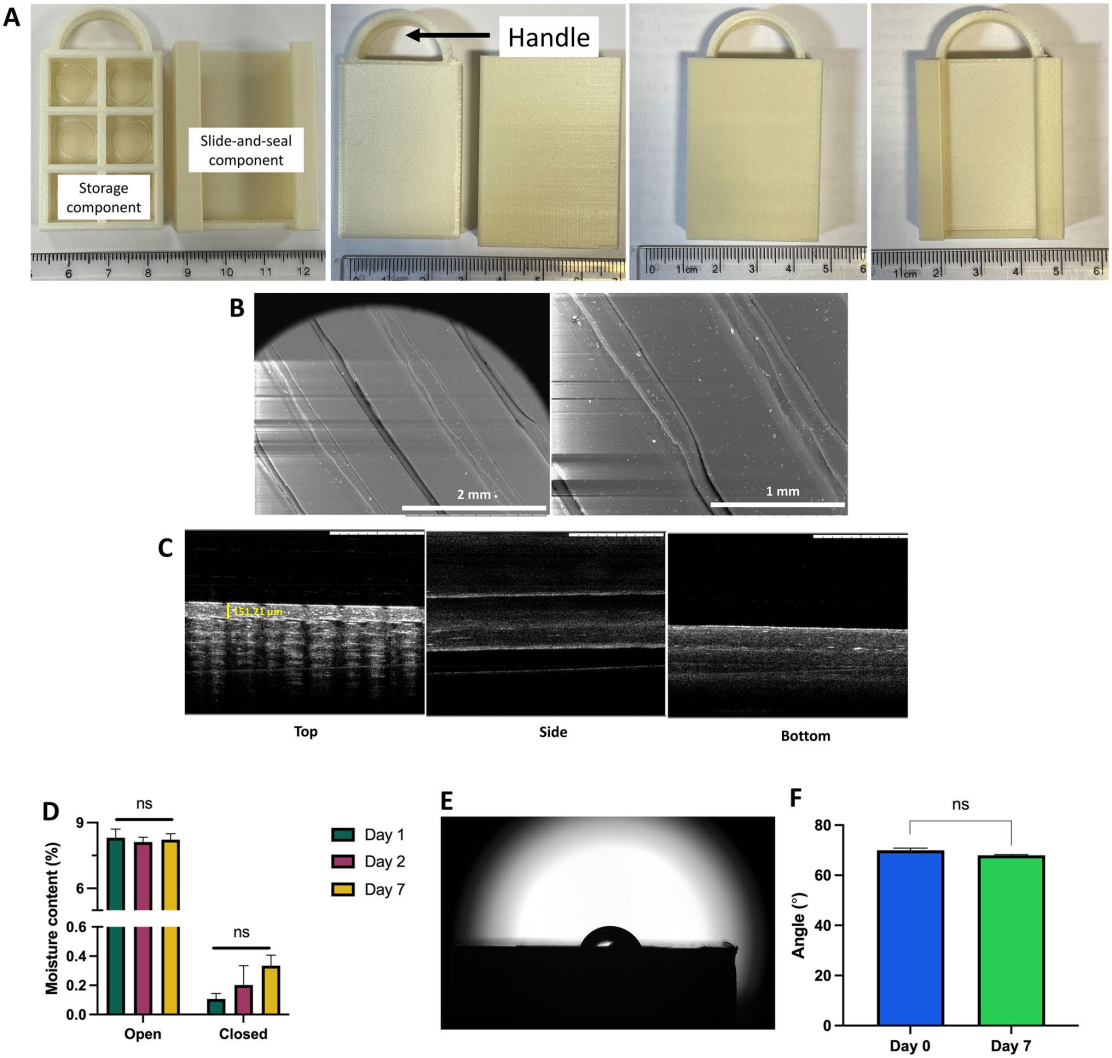
**A** Images of the slide and seal MAP-box, which was manufactured by FDM 3D-printing. **B** Surface of designed MAP-box using 50 × and 100 × magnification. **C** OCT images of MAP-box from different perspectives: top, side and bottom. **D** Moisture content of silica inside the designed MAP-box in open and closed system after exposed under 40 °C and 75% relative humidity at 1, 2 and 7 days (means + SD, *n* = 3). **E** Representative image for water contact angle measurement of the MAP-box surface. **F** Water contact angle measurement of MAP-box surface (means + SD, *n* = 3)

As shown in Fig. 5A, the MAP-box developed in the current work was fabricated using fused deposition modelling (FDM) 3D printing, which is a form of additive manufacturing. FDM fabricates the MAP-box in a sequential fashion, i.e., in a layer-by-layer manner by selectively depositing a melted thermoplastic polymer, in this instance PLA, in a predetermined path. The printed MAP-box consists of two components, namely a storage component and a slide-and-seal component. The storage component of the system could enable up to six patches to be stored per container while the slide-and-seal portion houses the storage component forming a closed system. The overall system is 3 cm wide and 4.5 cm long.

It is important to note that PLA is a biodegradable [36] and compostable polymer [37], and therefore, it will minimise the environmental impact of this approach. Moreover, the storage solution proposed in this work is reusable. Conventional healthcare plastic items are single-use, and this contributes to the global problem of plastic waste pollution [38].

This study used 3D-printing as it is a quick way to prepare prototypes. This technology has been extensively used to prepare medical devices and pharmaceutical preparations [39–44]. Moreover, it can be used on demand to prepare MAP-boxes or other medical devices on demand at the point-of-care [45]. In this work, we used FDM 3D-printing technology that is a low-cost technology. This enables its application in healthcare settings in developing countries [46]. However, if the MAP-box is required for larger amounts of patients, another suitable manufacturing technique such as injection moulding should be used rather than 3D-printing.

### Morphology and structure of MAP-box

SEM images show the surface of the MAP-box, as presented in Fig. 5B. Although some indentations can be seen, attributable to the 3D-printing process, OCT cross-sectional images of the top surface (Fig. 5C) confirm that these are only superficial. Indeed, when measured, they go down in only 0.15 mm from the surface, which represents only a 1.51% of the overall thickness of the MAP-box.

### Moisture permeability of MAP-box

As SEM studies showed some indentations on the MAP-box’s surface, which could have a detrimental effect on storage of MAPs due to the entrance of moisture, studies were conducted to assess this. Silica gel was placed on open MAP-boxes and stored in a 40 °C and 75% relative humidity, as the highest stress conditions used hereby. In Fig. 5D, it can be seen that after 24 h, the moisture content in silica gel (inferred from the weight increase) is around 9%, which does not change increase after a period of 2 or 7 days of incubation. Hence, studies were conducted within that time frame. Silica gel included in a closed MAP-box and exposed to the same conditions did have an apparent overall increasing trend, with around 0.3% moisture gain after 7 days. Nonetheless, no significant differences were seen in those values (*p* > 0.05).

### Water contact angle of MAP-box surface

To evaluate the hydrophilicity of the MAP-box, water contact angle was measured (Fig. 5E). It can be seen from Fig. 5F that before and after incubation of the MAP-box for 7 days, no significant differences were seen. As shown in the permeability study (Fig. 5D), only 1 day was enough to reach the maximum hydration of the silica gel and, hence, used as end time point for this study. Although a contact angle above 90 °C could guarantee to a certain extent the hydrophobicity of the material [47], the values found were slightly below. This could be attributed to the small indentations seen in the SEM studies which could favour the spreadability of water. Nonetheless, these seem to have a minimum effect on water permeability, as previously shown.

### Friability test of MAP inside the MAP-box

**Fig. 6:**
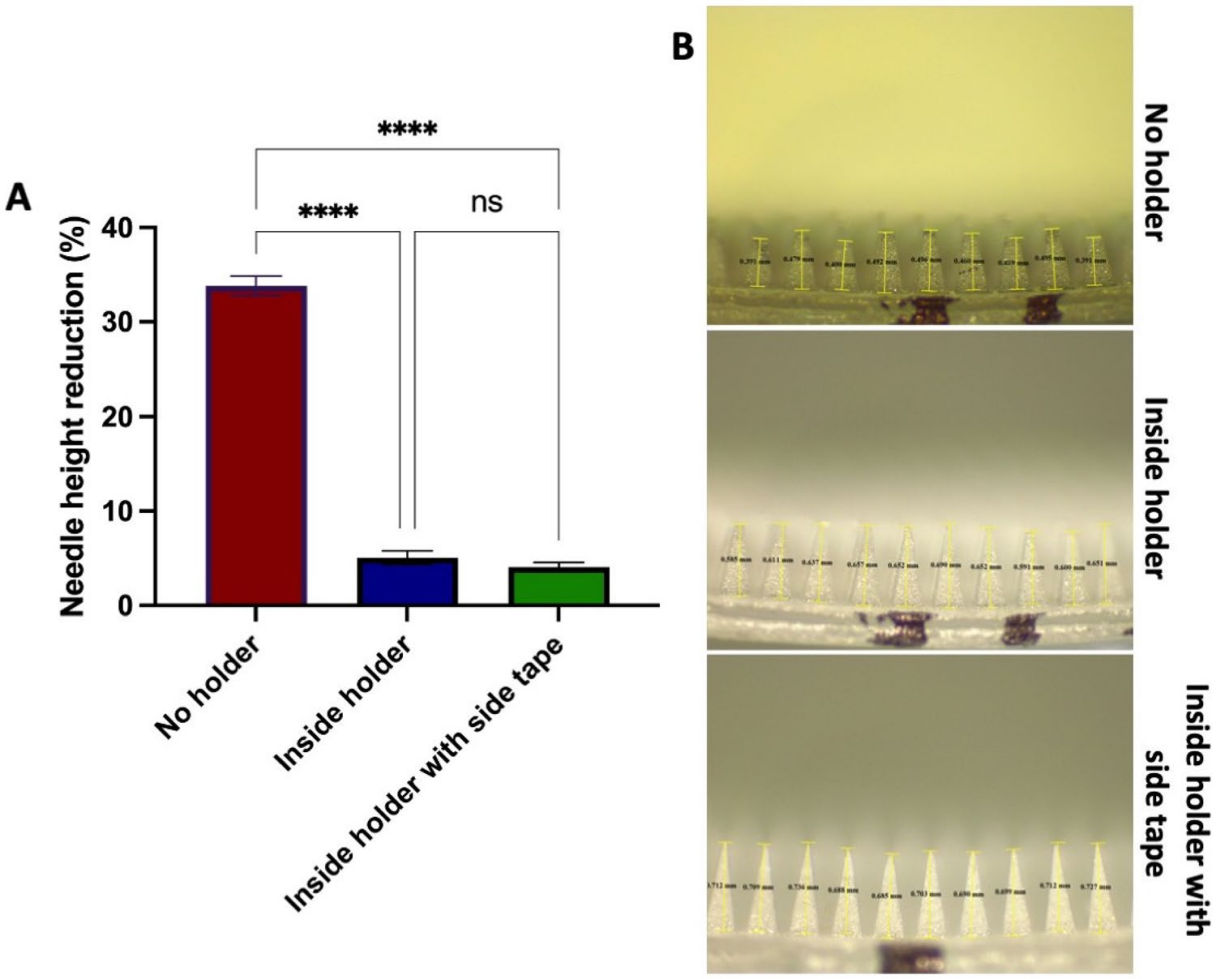
**A** Percentage of needle height reduction after friability test (means + SD, *n* = 20). **B** Representative images of needles after friability test

To explore the resistance of MAPs within the MAP-box upon potential stress forces, such as transportation, an adaptation of the friability test for tablets was used. As shown in Fig. 6A, MAPs placed in a falcon tube experienced a height decreased over 30% while MAPs within the MAP-box showed a height decrease below 5%. In addition, securing the patches with tape inside the MAP-boxes did not lead to significant differences in needle height (*p* > 0.05), confirming that the MAP-box keeps them in one place even during rotation. Light microscopy images allow to see that (Fig. 6B), as expected, the main effect of rotation is on MAP tips. It can be seen how they not only shrink but also lose their sharpness, which can certainly affect their insertion capabilities and hence, the overall transdermal drug delivery effect.

### Stability studies

The current work aimed to develop a novel MAP-box which may be suited for the storage of dissolving MAPs while still being easy to be used by patients. Upon the fabrication of both MAPs and the MAP-box, the formulations were stored at 5 °C and ambient humidity, 25 °C and 65% relative humidity and 40 °C and 75% relative humidity for 14 and 30 days. In all cases, MAPs were placed inside the containers before exposure to such conditions, using unpacked MAPs as controls. To evaluate the utility of the designed MAP-boxes in protecting MAPs over time, their drug content, height reduction and insertion depth were evaluated and used as indicative attributes of stability.

**Fig. 7:**
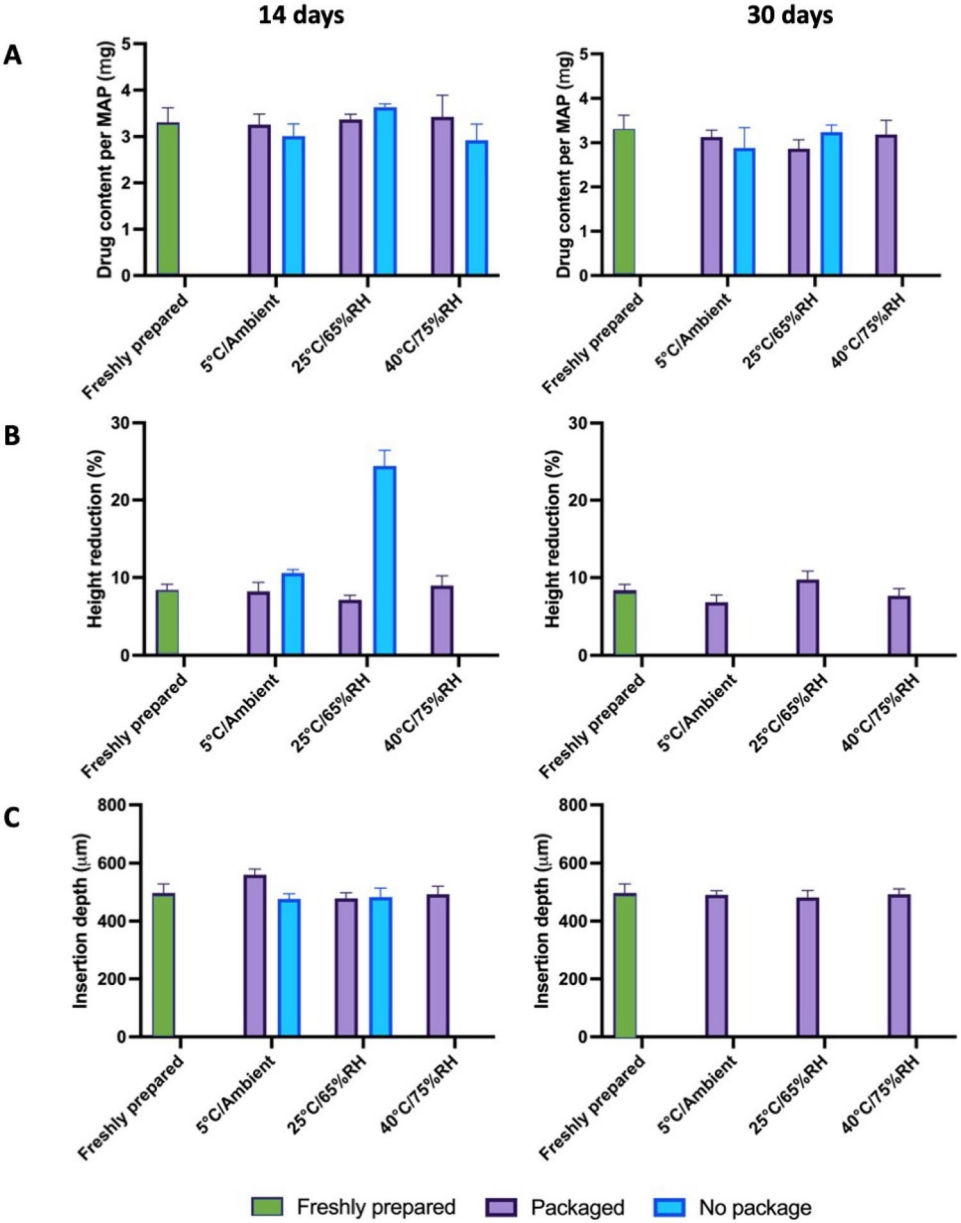
Stability studies of packed and unpacked donepezil-loaded MAPs exposed to different temperature and humidity conditions and their evaluation of **A** drug content (means + SD, *n* = 4), **B** height reduction (means + SD, *n* = 20) and **C** insertion depth (means + SD, *n* = 20). The absence of any bar charts for “no package” is due to the severe degradation of the MAP during storage which resulted in the ability of the patch to be characterised

As can be seen in Fig. 7A, no significant differences in drug content were seen after 14 days in both packed and unpacked MAPs (*p* > 0.05), as all of them stayed below the 115% of drug recovered. At 30 days, it was observed that complete API degradation occurred when MAPs were stored at 40 °C/75% RH. With the other conditions studied, no significant changes were seen in terms of drug load when not using a packaging (*p* > 0.05). With respect to height reduction and insertion depth (Fig. 7B, C), it was noted that, after 14 days, no significant differences were seen at 5 °C and ambient humidity (*p* > 0.05), whereas a significant increase in height reduction was seen when the needles were incubated at 25 °C/65% RH (*p* < 0.05). During the same time period and exposure at 25 °C/65% RH, unpacked MAPs displayed visual signs of moisture ingression into the polymeric patch, causing the MAPs to become soft as gelatin-like material, which made it impossible to run any insertion and mechanical characterisation. On day 30, the same observation was also noted for the unpacked MAPs. In contrast, all MAPs stored in the slide-and-seal primary package, regardless of the storage conditions, showed a similar height reduction of approximately 10% (*p* > 0.05), similar to the values initially found in newly fabricated MAPs. Based on the 30-day stability study conducted, it was apparent that the MAP-box developed endowed a protective effect on the MAPs when exposed to the different conditions over a long period of time (30 days), leading to no significant changes with respect to drug content, insertion depth and height reduction, which are key parameters for the successful insertion and later, transdermal delivery of compounds, proving overall the utility of the designed MAP-box in keeping MAPs protected for 30 days.

It has been reported that the main factors that cause drug instability and ultimately degradation are moisture, temperature and in some instances light [48]. With regard to this, meticulous consideration and design of MAP-box are pivotal during the development and scale-up of the pharmaceutical product. In the case of MAPs, particularly those developed from water-soluble polymers, the ingress of moisture through the packaging may result in plasticisation of the polymeric microneedle tip. This would soften the material and so impede the successful insertion of needles into the skin, turning the drug product useless for its intended use. It is often observed that in the case of a hygroscopic pharmaceutical product, the use of desiccants such as silica gels is commonly included in the primary packaging in order to generate a low relative humidity microenvironment within the package, conferring stability to the overall product [49].

In regards of potential protective solutions for MAPs, McAlister et al. [15] investigated the utility of Protect™ 470 foil in comparison to polyester foil in protecting hydrogel-forming MAP and amoxicillin sodium drug-containing reservoir [15]. In this work, McAlister et al. demonstrated the use of Protect™ 470 foil as a potential primary packaging for the storage of hydrogel-forming MAPs along with a drug-loaded reservoir, conferring stability to the whole system for up to 168 days. In comparison to the 3D printed PLA slide-and-seal package hereby developed, Protect™ 470 is manufactured from poly(propylene), poly(ethylene), foil and poly(ethylene), which have all been laminated together to form the primary packaging. Upon manufacture, poly(ethylene) may be heat-sealed to provide a moisture-free barrier to the stored pharmaceutical product [15]. In contrast, the use of the 3D-printed slide-and-seal MAP-box developed in the current work may help prevent the entrance of moisture into the MAPs.

It should be noted that the Food and Drug Administration (FDA) and its guideline for drug products Q1A(R2) suggest that both thermal stability and sensitivity to moisture should be tested, where storage conditions and lengths should be enough to cover the chain of storage, shipment and subsequent use of the drug product [50]. Although the present study was conducted for only 1 month, it confirms the physical integrity of MAPs (and its drug load) is maintained for at least 30 days. As mentioned earlier, the aim of the study was to prepare a MAP-box that could be potentially used to track MAP administration by the patient while providing protection to the MAPs from physical stress and environmental conditions. As the MAPs will be placed in the box just prepared for patient administration, the stability study was performed only over 30 days as the device is not designed to ensure long-term stability. While new research is currently undergoing in order to define the critical quality attributes and standardised tests required by regulatory agencies for the ultimate approval of such drug delivery systems, further studies could focus not only on longer stability studies but also on others pertained to the quality module of the Common Technical Document, namely container closure system and its effect on microbial stability as well as light protection to MAPs. Nonetheless, the studies hereby shown provide a proof of concept for the MAP-box designed, the low cost associated with its fabrication and ease of manufacture.

## Conclusion

In this work, we have evaluated the development of a slide-and-seal MAP-box which was prototyped using FDM 3D-printing. The slide-and-seal MAP-box was developed to store dissolvable MAPs which were fabricated from PVA and PVP and loaded with the antidementia drug, donepezil. The developed MAP was shown to have a high drug loading of 3 mg and was capable of completely dissolving in ex vivo skin following an application period of only 1 h. Upon application, the MAP was able to reach an insertion depth of 500 µm, which would allow them to pierce the *stratum corneum* and the viable skin layers, reaching later on the dermal microcirculation to be slowly delivered in a systemic fashion. Skin deposition studies showed that the system was able to deliver donepezil transdermally within 1 h of administration that culminated in an overall drug delivery efficiency of 70% after 24 h. Upon extensive pharmaceutical characterisation, MAPs were stored in the slide-and-seal primary package and stored under different storage conditions for up to 30 days. At pre-determined time points, the stability of MAPs was evaluated with respect to height reduction, skin insertion and drug content. It was observed that the simple slide-and-seal MAP-box was able to protect MAPs against moisture and heat thus preserving the overall integrity of the patch up to the 30 days. Therefore, this 3D-printed MAP-box prototype has been identified as a possibly suitable system to be used in storing MAPs for packaging and transport even in hot and humid conditions. Overall, this study highlights the importance of evaluating not just the pharmaceutical system developed but also the potential packaging that accompanies the product, as this would help in paving the way of MAPs into clinically available pharmaceutical drug products.

## Data Availability

The data is available from the corresponding author upon request.
